# Thermal Stability and Two-Step Devitrification of Melt-Spun Cr_16_Mn_16_Fe_16_Co_16_Ni_16_P_20_ High-Entropy Metallic Glass

**DOI:** 10.3390/ma19143034

**Published:** 2026-07-14

**Authors:** Krzysztof Ziewiec, Artur Błachowski, Krystian Prusik, Aneta Ziewiec

**Affiliations:** 1 Institute of Technology, University of the National Education Commission (UKEN), ul. Podchorążych 2, 30-084 Krakow, Poland; 2 Faculty of Geology, Geophysics and Environmental Protection, AGH University of Krakow, al. Adama Mickiewicza 30, 30-059 Krakow, Poland; artur.blachowski@agh.edu.pl; 3 Faculty of Science and Technology, Institute of Materials Engineering, University of Silesia (US), 75 Pułku Piechoty Street 1A, 41-500 Chorzów, Poland; krystian.prusik@us.edu.pl; 4 Faculty of Metals Engineering and Industrial Computer Science, AGH University of Krakow, al. Adama Mickiewicza 30, 30-059 Krakow, Poland; aziewiec@agh.edu.pl

**Keywords:** high-entropy metallic glass, melt spinning, phosphorus, thermal stability, devitrification, crystallization kinetics, Kissinger analysis, differential scanning calorimetry, X-ray diffraction, Mössbauer spectroscopy

## Abstract

**Highlights:**

- Cr_16_Mn_16_Fe_16_Co_16_Ni_16_P_20_ ribbon is fully amorphous after melt spinning.
- DSC reveals two devitrification peaks with E_a_ = 359.2 and 414.9 kJ/mol.
- Ex situ XRD suggests tentative Fe_3_P-type/FCC and MnNi-type/Co_2_P-type phase evolution.
- Mössbauer spectra show Fe-environment redistribution during heating.
- Devitrification requires higher kinetic barriers than simpler TM–P glasses.

**Abstract:**

The thermal stability and devitrification pathway of melt-spun high-entropy Cr_16_Mn_16_Fe_16_Co_16_Ni_16_P_20_ metallic glass were investigated using transmission electron microscopy/selected-area electron diffraction (TEM/SAED), differential scanning calorimetry (DSC), X-ray diffraction (XRD), and ^57^Fe Mössbauer spectroscopy. TEM/SAED confirmed an amorphous ribbon structure, with diffuse rings and radial maxima at k_1_ = 0.84799 nm^−1^ and k_2_ = 1.44459 nm^−1^. Non-isothermal DSC revealed two exothermic events, Peak I at ~716–752 K and Peak II at ~881–930 K, both shifting to higher temperatures with increasing heating rate. Kissinger analysis yielded apparent activation energies of Ea_1_ = 359.2 kJ/mol for Peak I and Ea_2_ = 414.9 kJ/mol for Peak II. Specimens heated in the DSC under argon at 20 K/min to selected target temperatures were examined ex situ. The XRD patterns are consistent with the onset of crystallization during Peak I, with reflections tentatively attributed to an Fe_3_P-type phase and an FCC solid solution. Peak II is associated with further phase evolution, including the development of reflections compatible with MnNi-type and Co_2_P-type phases. Because of peak overlap in this multicomponent alloy, the proposed phase sequence should be regarded as a plausible interpretation based on combined DSC, XRD, and Mössbauer evidence rather than as a uniquely resolved quantitative phase analysis. Mössbauer spectra reveal three paramagnetic Fe environments. With increasing DSC target temperature, the high-QS Fe3 component, representing a highly distorted Fe environment, decreases systematically, whereas the low-QS Fe1 component, associated with a more symmetric, nearly cubic Fe environment, becomes dominant. The high apparent activation energies indicate a larger effective kinetic barrier than in many simpler transition-metal–phosphorus amorphous alloys.

## 1. Introduction

Metallic glasses are alloys in which long-range crystalline order is suppressed during solidification or deposition. Their amorphous structure is typically identified by broad diffuse diffraction maxima rather than sharp Bragg reflections, while their thermal stability is commonly evaluated by differential scanning calorimetry and structural characterization after controlled heating. These approaches provide complementary information: DSC identifies thermally activated transformation events, whereas diffraction methods reveal the phase evolution associated with devitrification. The general principles of metallic-glass formation and crystallization provide the framework for interpreting such transformations in both conventional and chemically complex amorphous alloys [1,2].

Phosphorus has long been recognized as an effective glass-forming element in transition-metal alloys. Classical studies on Ni–P demonstrated that amorphous structures can be produced by electrodeposition or thin-film preparation and can subsequently crystallize during thermal treatment [3,4,5]. The formation and properties of amorphous Fe–C–P, Ni–Pd–P, Fe–Pd–P, Co–P, and Co–Ni–P alloys or films were also documented, confirming that phosphorus promotes chemically disordered, non-crystalline structures in different transition-metal systems [6,7,8,9,10]. These results provide the background for extending TM–P glass formation from simple binary and ternary alloys to multicomponent compositions such as Cr–Mn–Fe–Co–Ni–P.

In parallel, recent interest has focused on compositionally complex alloys, including high-entropy alloys and high-entropy metallic glasses, where several principal metallic elements are combined in near-equiatomic or otherwise highly concentrated proportions. In high-entropy metallic glasses, glass-forming ability, thermal stability, and crystallization behavior are commonly discussed in relation to chemical complexity, atomic-size mismatch, configurational entropy, and the competition between several possible crystalline products [11]. Compared with classical binary or ternary transition-metal–phosphorus metallic glasses, devitrification in high-entropy metallic glasses may involve more complex and frequently multistep phase evolution, because crystallization requires chemical partitioning among several transition-metal elements. Mechanistic discussions of devitrification in metallic glasses emphasize that crystallization may proceed through multiple exothermic events, reflecting an initial crystallization event during devitrification followed by further phase evolution at higher temperatures [12,13].

In this work, we investigate a melt-spun Cr_16_Mn_16_Fe_16_Co_16_Ni_16_P_20_ ribbon, where phosphorus is the only glass-forming element added to a near-equiatomic Cr–Mn–Fe–Co–Ni transition-metal matrix. The composition was selected to combine a chemically complex high-entropy-type metallic matrix with 20 at.% P, i.e., a phosphorus content comparable to that commonly used to stabilize amorphous transition-metal–phosphorus alloys. Building on our previous report on cooling-rate effects in this system [14], we provide a correlation between calorimetric signatures and structural/phase evolution by combining TEM/SAED, DSC, XRD after DSC heating to selected temperatures, and Mössbauer spectroscopy. The activation energies associated with the two crystallization events are evaluated using the Kissinger approach, which relates peak temperatures to heating rates in non-isothermal transformations [15]. The central aim is to correlate the two-step calorimetric devitrification behavior with phase evolution inferred from ex situ XRD and with local Fe-environment changes revealed by Mössbauer spectroscopy, thereby proposing a plausible devitrification pathway in a phosphorus-stabilized, multicomponent high-entropy metallic glass.

## 2. Materials and Methods

The Cr_16_Mn_16_Fe_16_Co_16_Ni_16_P_20_ alloy was synthesized by arc melting a 15 g batch prepared from elemental powders of Mn, Cr, Fe, Ni, and Co (99.95% purity) and phosphorus (98% purity). The powders (supplier: Onyxmet Tomasz Olszewski, Olsztyn, Poland) were thoroughly premixed, cold-compacted into a pellet, and melted under high-purity argon (99.999%). Before each melting cycle, the chamber was purged three times by evacuation to 10^−1^ Pa and refilling with argon to approximately 10^5^ Pa, followed by melting a 2 g titanium getter to further purify the atmosphere. Melting was performed in a custom arc furnace (Institute of Technical Sciences, University of the National Education Commission, Kraków, Poland) using a non-consumable tungsten electrode (4 mm diameter, tip angle 45–60°) at an arc current of 100 A. To ensure chemical homogeneity, the ingot was remelted seven times; each cycle lasted ~7 s, the ingot was inverted between cycles, and the surface was mechanically cleaned before remelting. After the final melting, the composition and homogeneity were verified by scanning electron microscopy coupled with energy-dispersive X-ray spectroscopy (SEM/EDS) using a JSM-6610LV scanning electron microscope (JEOL Ltd., Akishima, Tokyo, Japan) equipped with an X-Max 20 mm^2^ EDS detector (Oxford Instruments NanoAnalysis, High Wycombe, UK), using multiple measurement points on the surface and cross-section.

Amorphous ribbons were produced by melt spinning using a melt spinner manufactured by Artvac-Plus (Zabierzów, Poland). The wheel linear speed was 33 m/s, yielding cooling rates on the order of 10^5^–10^6^ K/s and a ribbon thickness of approximately 20 µm. These processing settings were kept identical to those used in the previous work to enable direct comparison of thermal and structural evolution.

Differential scanning calorimetry was carried out under flowing high-purity argon using a NETZSCH STA 449 F3 Jupiter simultaneous thermal analyzer (NETZSCH-Gerätebau GmbH, Selb, Germany). Heating runs were recorded for the as-quenched ribbon at β = 2, 5, 10, 20, and 40 K/min (exo up). Peak temperatures for both exothermic events were used for kinetic evaluation by the Kissinger method.

Phase evolution during devitrification was examined by X-ray diffraction (XRD) using a Rigaku MiniFlex II diffractometer (Rigaku Corporation, Tokyo, Japan). XRD patterns were collected for the as-spun ribbon and for specimens previously heated in the DSC instrument under flowing high-purity argon. The specimens were heated at 20 K/min to selected target temperatures corresponding to the temperature ranges of Peak I and Peak II and then cooled to room temperature before ex situ XRD analysis. Diffraction scans were collected in the 2θ range of 10–120° with a step size of 0.02°. The diffractometer was operated at 30 kV and 15 mA.

Transmission electron microscopy (TEM) and selected-area electron diffraction (SAED) were used to confirm the amorphous structure of the as-spun ribbon. Thin-foil specimens were prepared from fragments of the as-spun melt-spun ribbon. The specimens were mechanically pre-thinned by grinding and dimpling and then ion-milled to electron transparency using a Model 691 Precision Ion Polishing System (PIPS; Gatan Inc., Pleasanton, CA, USA). Final thinning was performed by low-angle Ar-ion milling at 5 keV until perforation. TEM observations were carried out using a JEM-3010 transmission electron microscope (JEOL Ltd., Akishima, Tokyo, Japan) operated at an accelerating voltage of 300 kV, and images were recorded using an Orius SC200D CCD camera (Gatan Inc., Pleasanton, CA, USA). SAED patterns were acquired from at least five distinct regions of interest using selected-area apertures corresponding to calibrated specimen areas of approximately 400 and 1000 nm. The diffraction patterns were recorded at a camera length of 1000 mm. The camera constant was calibrated using an evaporated aluminium standard (Agar Scientific Ltd., Rotherham, UK). SAED patterns were evaluated for diffuse halos without discrete diffraction spots or sharp crystalline rings. Radial intensity profiles of the SAED patterns were obtained using ImageJ software (version 1.54d; National Institutes of Health, Bethesda, MD, USA) to determine the positions of characteristic diffuse maxima in reciprocal space.

Room-temperature ^57^Fe Mössbauer spectra were collected for the as-quenched ribbon and for specimens previously heated in the DSC to selected target temperatures. The measurements were performed in transmission geometry using a RENON MsAa-4 spectrometer (RENON, Kraków, Poland) equipped with a ^57^Co(Rh) source and an LND Kr-filled proportional detector. Absorbers were prepared from ribbon material by filing with a diamond file or grinding in an agate mortar to obtain powders with an areal density of ~10 mg/cm^2^. Spectra were processed with the MOSGRAF software suite (version 2.2, Institute of Nuclear Physics PAN, Kraków, Poland) using the transmission integral approximation. Isomer shifts are reported relative to α-Fe at room temperature, and reported uncertainties correspond to the last significant digit.

## 3. Results

**Figure 1:**
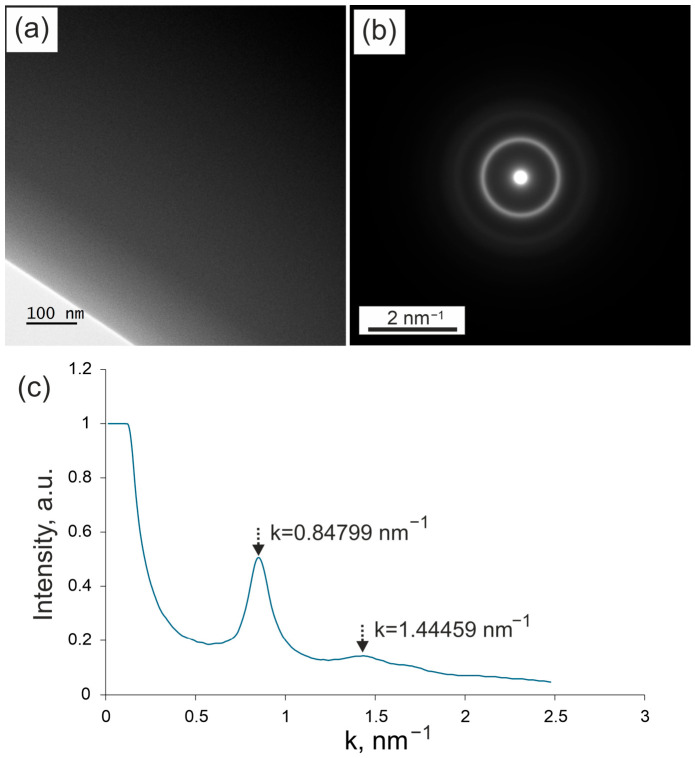
(**a**) Representative bright-field TEM micrograph of an ion-thinned thin foil prepared from the as-spun Cr_16_Mn_16_Fe_16_Co_16_Ni_16_P_20_ melt-spun ribbon, showing uniform contrast without visible crystalline grains. (**b**) Selected-area electron diffraction (SAED) pattern recorded from a representative region, exhibiting diffuse halos characteristic of a disordered atomic arrangement and no discrete diffraction spots or sharp crystalline rings. (**c**) Radial intensity distribution of the SAED pattern obtained using ImageJ, with broad maxima located at k = 0.84799 nm^−1^ and k = 1.44459 nm^−1^.

Figure 1 presents the TEM/SAED results for the as-spun melt-spun Cr_16_Mn_16_Fe_16_Co_16_Ni_16_P_20_ alloy ribbon. The TEM specimen was an ion-thinned thin foil prepared from the as-spun ribbon. At least five distinct regions of interest were examined, and the bright-field TEM micrograph and SAED pattern shown in Figure 1 were taken from a representative region of the specimen. The bright-field image reveals uniform contrast, with no visible crystalline grains. The corresponding SAED pattern consists of broad diffuse halos and does not display discrete diffraction spots or sharp crystalline rings characteristic of crystalline phases. The radial intensity distribution obtained from the SAED pattern using ImageJ shows two broad maxima at k = 0.84799 nm^−1^ and k = 1.44459 nm^−1^. The diffuse nature of the diffraction features and the absence of sharp reflections confirm the lack of long-range atomic order in the examined regions and verify the amorphous structure of the as-spun alloy, consistent with the room-temperature XRD pattern.

It should be noted that in our previous publication [14], slightly different k values were reported for the diffraction halos. However, a repeated and refined analysis of the SAED patterns performed in the present study yielded corrected wave-vector values, which are provided here. The updated values more accurately reflect the radial intensity distribution of the diffuse diffraction rings.

**Figure 2:**
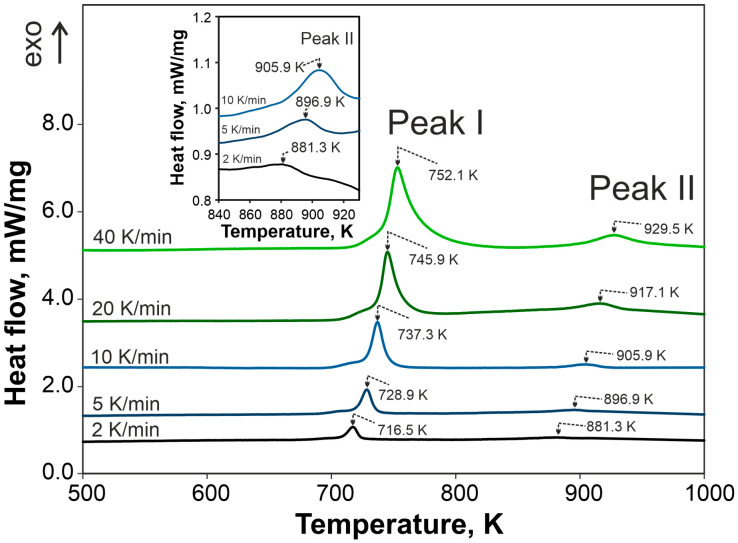
DSC heating curves of the melt-spun Cr_16_Mn_16_Fe_16_Co_16_Ni_16_P_20_ ribbon in the as-quenched amorphous state recorded at heating rates of 2, 5, 10, 20, and 40 K/min (exo up). Two exothermic peaks (Peak I and Peak II) related to crystallization are observed. The inset presents an enlarged view of Peak II for the three lowest heating rates, with an expanded vertical scale for clarity.

The DSC curves shown in Figure 2 reveal a two-stage crystallization process of the amorphous Cr_16_Mn_16_Fe_16_Co_16_Ni_16_P_20_ alloy during heating. The first exothermic peak (Peak I) appears in the temperature range of approximately 716–752 K, while the second peak (Peak II) is observed between about 881 and 930 K, depending on the heating rate. Increasing the heating rate results in a systematic shift in both peak temperatures toward higher values, consistent with a thermally activated crystallization process. No distinct glass-transition signal was resolved before the onset of crystallization under the applied DSC conditions. Therefore, Tg and the supercooled liquid region ΔTx could not be reliably determined. For this reason, the thermal stability and crystallization kinetics are discussed using the peak temperatures Tp of Peak I and Peak II. Peak I is relatively sharp and well-defined over the entire range of heating rates, whereas Peak II is broader and less intense, particularly at lower heating rates, which required magnification of the vertical scale in the inset. The peak temperatures of both events were used for the activation energy calculations.

**Figure 3:**
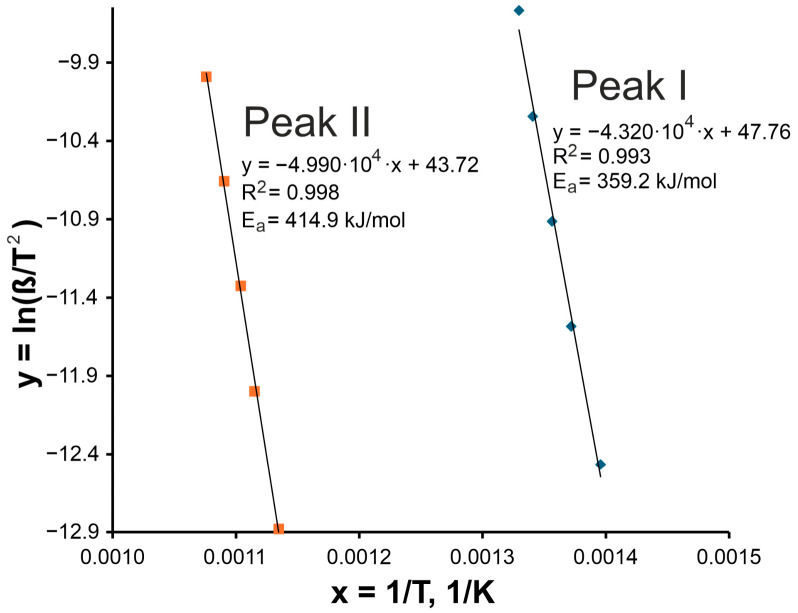
Kissinger plots for Peak I and Peak II constructed from DSC measurements of the amorphous Cr_16_Mn_16_Fe_16_Co_16_Ni_16_P_20_ alloy. Linear fits of ln(β/Tp^2^) versus 1/Tp are shown together with the regression equations and correlation coefficients. The apparent activation energies determined from the slopes are 359.2 kJ/mol for Peak I and 414.9 kJ/mol for Peak II.

Figure 3 shows the Kissinger plots, i.e., the linear dependence of ln(β/Tp^2^) on 1/Tp, used to determine the apparent activation energy for both devitrification events. Excellent linear fits were obtained for Peak I and Peak II (R^2^ = 0.993 and 0.998, respectively). The apparent activation energies derived from the slopes are 359.2 kJ/mol for Peak I and 414.9 kJ/mol for Peak II. Thus, the higher-temperature Peak II exhibits a higher apparent activation energy than the lower-temperature Peak I. These values should be treated as peak-temperature-based effective kinetic barriers for complex devitrification events, not as activation energies of single elementary diffusion processes. Their physical meaning is discussed below in relation to the phase evolution observed by XRD and the redistribution of Fe local environments detected by Mössbauer spectroscopy.

**Figure 4:**
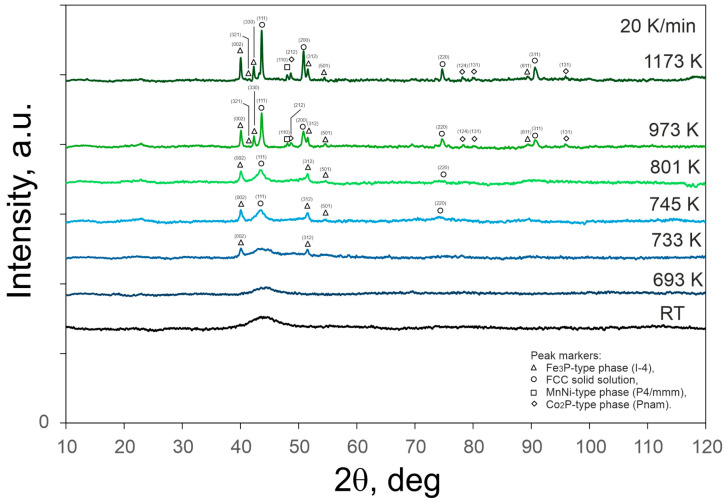
XRD patterns of the Cr_16_Mn_16_Fe_16_Co_16_Ni_16_P_20_ melt-spun ribbon recorded for the as-quenched state (RT) and for specimens previously heated in the DSC to 693, 733, 745, 801, 973, and 1173 K at a heating rate of 20 K/min under argon. The patterns illustrate progressive crystallization of the initially amorphous alloy. Peak markers indicate reflections tentatively attributed to: △ Fe_3_P-type phase (I-4), ○ FCC solid solution, □ MnNi-type phase (P4/mmm), and ◇ Co_2_P-type phase (Pnam).

Figure 4 shows the XRD patterns of the as-spun ribbon and of the specimens heated in the DSC to selected target temperatures. The diffraction pattern recorded at room temperature (RT) for the as-spun ribbon exhibits a broad diffuse maximum without sharp Bragg reflections, confirming its amorphous character. After DSC heating to 693 K and subsequent cooling to room temperature, the pattern remains predominantly amorphous, showing only a slight narrowing of the broad maximum. At 733 and 745 K, the first crystalline reflections appear. These reflections are consistent with the onset of devitrification and are tentatively attributed mainly to an Fe_3_P-type phase (tetragonal, space group I-4, No. 82, PDF 04-007-1524) and an FCC solid solution (cubic structure, PDF 01-071-8288). The FCC contribution should be interpreted as an FCC solid solution rather than pure fcc-Fe, because the exact chemical composition of this phase cannot be determined from the present laboratory XRD data alone.

Further DSC heating to 801 and 973 K leads to the emergence and growth of additional reflections compatible with MnNi-type phase (tetragonal, space group P4/mmm, No. 123, PDF 04-003-5437) and Co_2_P-type phase (orthorhombic, space group Pnam, No. 62, PDF 04-003-3925), reflecting the development of a multiphase crystalline structure. At 1173 K, sharper and better-defined diffraction peaks from the proposed phase assemblage are observed, indicating advanced devitrification of the amorphous matrix. Because several reflections overlap in this multicomponent alloy, the phase assignment should be treated as qualitative and tentative.

**Figure 5:**
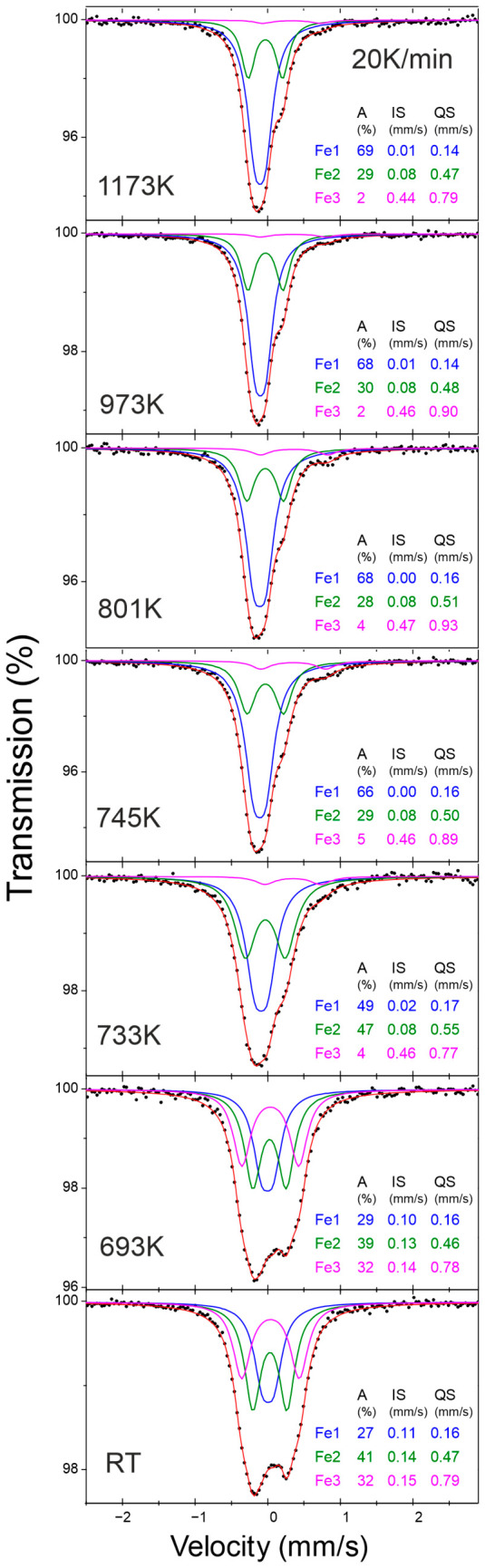
Room-temperature ^57^Fe Mössbauer spectra of the Cr_16_Mn_16_Fe_16_Co_16_Ni_16_P_20_ alloy in the as-quenched state (RT) and for specimens previously heated in the DSC to 693, 733, 745, 801, 973, and 1173 K at a heating rate of 20 K/min. Experimental data (black points) are shown together with the total fit (red line) and three paramagnetic subspectra: Fe1 (blue), Fe2 (green), and Fe3 (magenta), representing phenomenological local Fe environments. The relative spectral contributions (A), isomer shifts (IS), and quadrupole splittings (QS) are indicated in the figure.

Figure 5 presents the room-temperature ^57^Fe Mössbauer spectra of the as-quenched alloy and of the specimens previously heated in the DSC to selected target temperatures. The room-temperature Mössbauer spectrum of the as-spun sample consists of three paramagnetic subspectra with different quadrupole splittings, indicating distinct local environments of Fe atoms in the amorphous structure. The Fe3 component, which exhibits the highest QS value, accounts for approximately 32% of the total spectral area, suggesting a highly distorted local Fe environment. After DSC heating to 693 K followed by cooling to room temperature, all three components remain clearly distinguishable, with comparable contributions of Fe1 and Fe2 and a still significant Fe3 fraction. At 733 K, a pronounced redistribution of the relative spectral areas is observed: the Fe1 component, characterized by the smallest QS and associated with an Fe environment close to cubic symmetry, increases markedly, whereas the high-QS Fe3 component decreases significantly. This indicates progressive rearrangement of local Fe environments associated with the onset of crystallization. Further DSC heating to 745–801 K continues this trend, with the Fe1 contribution increasing up to ~68% and Fe3 decreasing to ~4%, reflecting increasing structural ordering around Fe atoms. At 973 and 1173 K, the spectra are dominated by Fe1 (~68–69%), while Fe2 remains nearly constant (~28–30%) and Fe3 is reduced to a minor fraction (~2%). The gradual decrease in the high-QS component and stabilization of Fe1 with increasing target temperature indicate progressive devitrification and a reduction in highly distorted Fe environments.

The FWHM values remain within a relatively narrow range of approximately 0.26–0.35 mm/s for all investigated states. No systematic line broadening is observed after DSC heating to the selected temperatures. The slightly higher linewidths in the as-spun sample may reflect a broader distribution of local Fe environments typical of the amorphous state, whereas the relatively stable linewidths after DSC heating indicate that crystallization is accompanied mainly by changes in the relative fractions of Fe environments rather than by pronounced broadening of the hyperfine-parameter distribution.

## 4. Discussion

The melt-spun Cr_16_Mn_16_Fe_16_Co_16_Ni_16_P_20_ ribbon is amorphous in the as-spun state, as indicated by diffuse SAED rings and the absence of sharp Bragg reflections in the room-temperature XRD pattern. This behavior is consistent with the long-established role of phosphorus as an efficient glass-forming element in transition-metal alloys. Early studies documented amorphous Ni–P and related transition-metal–phosphorus alloys or films, including Fe–C–P, Ni–Pd–P, Fe–Pd–P, Co–P, and Co–Ni–P systems, establishing phosphorus as a metalloid that favors chemically disordered, non-crystalline structures over a broad range of transition-metal compositions [3,4,5,6,7,8,9,10]. The present Cr–Mn–Fe–Co–Ni–P alloy extends this classical TM–P glass-forming tendency to a multicomponent, near-equiatomic transition-metal matrix. From the perspective of high-entropy metallic glasses, the present alloy represents a phosphorus-containing multicomponent metallic glass in which thermal stability is governed not only by the glass-forming role of phosphorus, but also by the chemical complexity of the Cr–Mn–Fe–Co–Ni matrix. In the present DSC curves, no distinct glass-transition signal was resolved before crystallization; therefore, Tg and the supercooled liquid region ΔTx could not be reliably determined. For this reason, the thermal stability of this alloy is discussed mainly in terms of crystallization peak temperatures, their heating-rate dependence, apparent activation energies, and ex situ phase evolution. The observed two-step devitrification behavior is consistent with the expected complexity of crystallization in high-entropy metallic glasses, where phase selection and chemical partitioning may proceed through successive transformation stages rather than through a single crystallization event.

The two exothermic events observed by DSC can be interpreted as two successive stages of devitrification. Peak I (~716–752 K) corresponds to the onset of crystallization in the amorphous matrix during heating. This interpretation is supported by ex situ XRD, since the first crystalline reflections appear at approximately 733–745 K. These reflections are tentatively attributed mainly to an Fe_3_P-type phase and an FCC solid solution. Thus, the first DSC event is not simply structural relaxation, but marks the beginning of crystallization within the amorphous matrix. This interpretation is consistent with general devitrification concepts in metallic glasses [12,13]. In the present alloy, Mössbauer spectroscopy provides complementary local evidence: the decrease in the high-QS Fe3 component, representing highly distorted Fe environments, and the increase in the low-QS Fe1 component, associated with a more symmetric Fe environment close to cubic symmetry, indicate a redistribution of local Fe environments accompanying the first devitrification stage.

It should be noted that TEM/SAED was used in the present work to verify the amorphous structure of the as-spun ribbon, whereas the structural evolution after DSC heating was followed by ex situ XRD and Mössbauer spectroscopy. Direct TEM characterization of the devitrified states would require a separate specimen-preparation route, because the DSC-heated melt-spun ribbons become mechanically fragile after devitrification and the expected crystallization products are nanometric. Conventional SEM/EDS is also not sufficient for reliable phase identification of such nanoscale devitrification products. Therefore, direct nanoscale morphology, local chemistry, and quantitative phase-fraction analysis of the devitrified specimens are outside the scope of the present work and will require future dedicated HR-TEM/SAED or STEM-EDS studies of specially prepared specimens.

Peak II (~881–930 K) occurs after the alloy has already partially crystallized. It therefore corresponds to further phase evolution rather than to the initial onset of crystallization. Ex situ XRD shows that, upon further heating, additional reflections compatible with MnNi-type and Co_2_P-type phases develop and increase in intensity, while the material evolves toward a multiphase crystalline state. In this sense, Peak II can be associated with the second devitrification stage, involving further growth of crystalline regions, development of additional phases, and chemical partitioning within the partially crystallized material [12,13].

The Kissinger analysis gives apparent activation energies and should therefore be interpreted as a measure of the effective kinetic barrier for each devitrification event, rather than as the activation energy of a single elementary process. In metallic glasses, such values may include contributions from nucleation, diffusion, interface formation, chemical partitioning, and crystal growth [12,13,15]. This limitation is particularly important for the present multicomponent alloy, where each DSC peak represents a complex transformation involving phase selection and redistribution of several transition-metal elements.

A full isoconversional analysis, such as KAS, Ozawa, or Vyazovkin treatment, was not applied in the present work because the analysis was based on peak temperatures of two non-isothermal DSC events. Peak II is broad and comparatively weak at lower heating rates, and the crystallization process is multistep rather than a single isolated reaction. Under these conditions, extracting reliable conversion-dependent activation energies would require a separate deconvolution-based kinetic study. Therefore, the present Kissinger analysis is used as a comparative estimate of the effective kinetic barriers associated with Peak I and Peak II.

In the present alloy, Ea_2_ for Peak II (414.9 kJ/mol) is higher than Ea_1_ for Peak I (359.2 kJ/mol). This indicates that the higher-temperature transformation remains kinetically demanding, most likely because it involves further chemical redistribution and development of a more complex crystalline phase assemblage in an already partially crystallized material. Peak I, in contrast, marks the onset of crystallization during devitrification, with the appearance of reflections tentatively attributed to Fe_3_P-type and FCC crystalline products.

Taken together, DSC identifies two thermally activated events, ex situ XRD reveals the progressive appearance of crystalline reflections, and Mössbauer spectroscopy shows that crystallization is accompanied by a redistribution of local Fe environments. The strongest Mössbauer change occurs around the first devitrification stage, where the high-QS Fe3 component decreases abruptly and the low-QS Fe1 component becomes dominant. This indicates that the onset of crystallization is accompanied by a pronounced reduction in highly distorted Fe environments and an increase in more symmetric Fe environments.

The Fe1, Fe2, and Fe3 subspectra should be interpreted as phenomenological local Fe environments rather than as unique one-to-one assignments to individual crystallographic phases. In a partially devitrified multicomponent alloy, several Fe-containing local environments may contribute to overlapping Mössbauer components, while some phases detected by XRD may contain only limited amounts of Fe or Fe substituted at different crystallographic sites. Therefore, assigning each Mössbauer component uniquely to a specific XRD phase would not be justified.

For specimens heated to higher target temperatures and subsequently measured at room temperature, the spectra are still described without a resolved magnetic hyperfine component, and the linewidths remain relatively stable. The absence of a resolved magnetic component does not contradict crystallization; rather, it indicates that no dominant, well-resolved magnetically ordered Fe-rich phase is detected in the adopted fitting model. Any Fe-containing crystalline contribution may be minor, chemically substituted, broadened, or overlapped with the dominant paramagnetic Fe environments. Thus, Mössbauer spectroscopy supports the redistribution of local Fe environments during devitrification, whereas the exact phase-specific assignment remains tentative.

The magnitude of E_a1_ and E_a2_ can be placed in context by comparison with simpler amorphous alloys in which phosphorus is the main glass-forming element. In binary Ni–P metallic glass, Lu and Wang reported two activation energies associated with different parts of the crystallization process: about 324 kJ/mol for nucleation-related kinetics and about 180 kJ/mol for growth-related kinetics [16]. This distinction is important because it shows that the reported value of E_a_ depends on which stage of crystallization is being analyzed.

Further reference points are provided by amorphous Fe–Ni–P deposits. Expressed in atomic percent, the alloys with P close to ~14 at.% (Fe_72.85_Ni_13.50_P_13.65_ and Fe_3.92_Ni_81.96_P_14.12_) show activation energies of ~297–304 kJ/mol, whereas alloys with higher phosphorus content of ~20–23 at.% P (Fe_73.23_Ni_6.97_P_19.80_ and Fe_43.88_Ni_33.05_P_23.07_) show lower values of ~195–198 kJ/mol [17]. Gao reported similarly lower values for electrodeposited Ni_69_Fe_8_P_23_, where the activation energies of successive exothermic events ranged from 111.6 to 253.6 kJ/mol [18]. In Ni–Sn–P amorphous coatings, Yu et al. obtained effective crystallization activation energies of 275.68 kJ/mol in the as-deposited state and 239.75 kJ/mol after heavy plastic deformation [19]. A direct Fe–P reference is provided by Zhang et al., who reported 148.0 kJ/mol at T_x_ and 144.5 kJ/mol at T_p_ for electrodeposited amorphous Fe_81.5_P_18.5_; adding a small amount of Yb increased these values to 224.7 and 255.0 kJ/mol [20].

These literature values are lower than the 359–415 kJ/mol obtained for the present Cr_16_Mn_16_Fe_16_Co_16_Ni_16_P_20_ alloy. Although the comparison is necessarily qualitative because of differences in composition, preparation route, and analysis method, it suggests that devitrification in the multicomponent TM–P glass involves a higher effective kinetic barrier than in many simpler phosphorus-containing amorphous alloys. This difference is consistent with the need for more extensive chemical partitioning and phase selection in the Cr–Mn–Fe–Co–Ni–P system.

On this basis, the high Ea values should be treated as effective barriers for a coupled transformation sequence rather than as direct diffusion energies of individual elements. Peak I marks the onset of crystallization during devitrification, including nucleation, interface formation, and initial chemical partitioning. Peak II proceeds through further redistribution of components and development of a more complex phase assemblage. Therefore, the Ea_2_ > Ea_1_ relation is physically consistent with a higher effective barrier for the higher-temperature phase-evolution event in a partially crystallized material [12,13,15].

The DSC peaks can therefore be interpreted as follows: Peak I corresponds to the first devitrification stage, associated with the onset of crystallization in the amorphous ribbon and the appearance of reflections tentatively attributed to an Fe_3_P-type phase and an FCC solid solution, accompanied by the strongest redistribution of Fe local environments. Peak II corresponds to further phase evolution during devitrification; additional reflections compatible with MnNi-type and Co_2_P-type phases develop, while the Mössbauer spectra remain paramagnetic and the linewidths remain comparatively stable. This supports a devitrification pathway governed by successive phase evolution and chemical redistribution rather than by a single abrupt structural transformation. However, because of peak overlap in the XRD patterns of this multicomponent alloy, the proposed phase sequence should be regarded as a plausible interpretation rather than as a uniquely resolved quantitative phase analysis [12,13].

## 5. Conclusions

The melt-spun Cr_16_Mn_16_Fe_16_Co_16_Ni_16_P_20_ ribbon is amorphous in the as-spun state, as indicated by TEM/SAED observations and by the absence of sharp Bragg reflections in the room-temperature XRD pattern.

DSC reveals a two-step devitrification process, with Peak I at ~716–752 K and Peak II at ~881–930 K. The peak temperatures increase with heating rate, confirming the thermally activated character of both transformations. No distinct glass-transition signal was resolved before crystallization under the applied DSC conditions; therefore, Tg and ΔTx could not be reliably determined.

Kissinger analysis gives high apparent activation energies of 359.2 kJ/mol for Peak I and 414.9 kJ/mol for Peak II. These values should be regarded as effective kinetic barriers for complex devitrification events rather than as activation energies of single elementary processes.

XRD results obtained after DSC heating to selected temperatures are consistent with the onset of crystallization during Peak I, with reflections tentatively attributed to an Fe_3_P-type phase and an FCC solid solution. Peak II is associated with further phase evolution, including the development of reflections compatible with MnNi-type and Co_2_P-type phases, leading to a complex multiphase crystalline state after heating to 1173 K. Because of peak overlap in this multicomponent alloy, the proposed phase sequence should be regarded as a plausible interpretation rather than as a uniquely resolved quantitative phase analysis.

Room-temperature Mössbauer spectroscopy shows three paramagnetic subspectra representing phenomenological local Fe environments. With increasing DSC target temperature, the high-QS Fe3 component, associated with highly distorted Fe environments, decreases systematically, whereas the low-QS Fe1 component, associated with a more symmetric Fe environment close to cubic symmetry, becomes dominant. This confirms a pronounced redistribution of local Fe environments during devitrification. The Mössbauer components should not be interpreted as unique one-to-one assignments to individual crystalline phases.

Compared with many simpler phosphorus-containing amorphous alloys, Cr_16_Mn_16_Fe_16_Co_16_Ni_16_P_20_ exhibits higher apparent activation energies, suggesting a larger effective kinetic barrier for crystallization during devitrification in this multicomponent TM–P glass. The combined DSC, XRD, and Mössbauer results support a multi-step devitrification pathway governed by successive phase formation and chemical redistribution.

## Data Availability

The original contributions presented in this study are included in the article. Further inquiries can be directed to the corresponding author.
